# ERCC1, RRM1 and TUBB3 mRNA expression on the tumor response and overall survival of non-small cell lung cancer treated with platinum-based chemotherapy

**DOI:** 10.12669/pjms.306.5768

**Published:** 2014

**Authors:** Hongwei Qiao, Xiaoping Huang, Hua Guo, Yan Liu, Chunyan Yue

**Affiliations:** 1 Hongwei Qiao, The Second Respiratory Medicine Department, Xinxiang Central Hospital, Xinxiang, China.; 2 Xiaoping Huang, The Second Respiratory Medicine Department, Xinxiang Central Hospital, Xinxiang, China.; 3 Hua Guo, The Second Oncology Department, Xinxiang Central Hospital, Xinxiang, China.; 4 Yan Liu, Department of Tumor Surgery, Xinxiang Central Hospital, Xinxiang, China.; 5 Chunyan Yue, The Second Oncology Department, Xinxiang Central Hospital, Xinxiang, China.

**Keywords:** ERCC1, RRM1, TUBB3; mRNA, non-small cell lung cancer, Tumor response, Overall survival

## Abstract

***Objective: ***We aimed to analyze the expression of ERCC1, RRM1 and TUBB3 in 305 patients with advanced non-small cell lung cancer (NSCLC) and investigate whether these genes can be used as biomarkers for predicting tumor response and clinical outcome.

***Methods: ***Total 305 patients with unresectable and locally advanced NSCLC were collected between January 2007 and December 2008. cDNA of ERCC1, RRM1 and TUBB3 was isolated by a fluorescence-based real-time detection method.

***Results:*** All the patients were followed up until December 2012. One hundred seventy five patients showed good response and 130 patients showed poor response to chemotherapy. 126 patients died and 166 patients showed progressive disease during the follow-up period. The median levels of ERCC1, RRM1 and TUBB3 mRNA were 0.53±0.13, 0.31±0.15 and 0.18±0.16, respectively. We found that patients with low ERCC1 expression showed a significantly higher rate of good tumor response, and the adjusted OR (95% CI) was 2.16(1.32-3.45). By Cox regression analysis. We also found that low ERCC1 expression level were correlated with longer overall survival of NSCLC patients, with the adjusted HR (95% CI) was 2.15 (1.26–3.35).

***Conclusion:*** This study showed that ERCC1 mRNA expression can not affect the response to chemotherapy and clinical outcome of advanced non-small cell lung cancer (NSCLC) patients.

## INTRODUCTION

Lung cancer is the leading cause of cancer-related deaths for both males and females worldwide, and non-small cell lung cancer (NSCLC) accounts for about 70%~85% of all lung cancer cases.^1^ It is estimated that more than 60% of NSCLC patients showed unresectable advanced disease when they were diagnosed. Combined modality therapy has become the standard method in treatment of NSCLC, and chemotherapy has become the integral part therapy for NSCLC.^2^ For patients with advanced stage NSCLC, chemotherapy could improve the quality of life and prolong survival time in patients with advanced NSCLC. It is reported that platinum-based doublet chemotherapy is the standard therapy for patients with advanced NSCLC.^3^^,^^4^ Even the advanced therapy, the 2-year overall survival rate for unresectable NSCLC is about 15%. 

Although great efforts contribute to improve the monotherapy or combinational chemotherapy regimens, the clinical outcome of NSCLC is still not satisfactory.^5^^-^^7^ It is estimated that individual chemotherapy by molecular biomarkers can play a role in improving the survival time of NSCLC treatment.^7^^,^^8^ Biomarkers-based molecularly chemotherapy play an important role in improving the overall survival of advanced NSCLC. It is well known that epidermal growth factor receptor contributes to a better tumor response to chemotherapy. Similarly, excision repair cross-complementing gene 1 (ERCC1) and ribonucleotde reductase M1 (RRM1) and *β*-tubulin III (TUBB 3) expression are associated with therapeutic efficacy of several chemotherapy regimens, such as platinum, gemcitabine and docetaxel.^9^^-^^11^

However, few studies have investigated the effect of ERCC1, RRM1 and β-tubulin III(TUBB3) expression on the clinical outcome of NSCLC or the association between ERCC1, RRM1 and TUBB3 and clinical characteristics.^12^^,^^13^ In the current study, we aimed to analyze the expression of ERCC1, RRM1 and TUBB3 in 305 patients with advanced NSCLC, and investigated whether these genes can be used as biomarkers for predicting tumor response and clinical outcome.

## METHODS

***Patients and samples*** **: **A total of 305 patients with unresectable and locally advanced NSCLC who were admitted at Xinxiang Central Hospital between January 2007 and December 2008 were recruited in our study. All patients were pathologically or radiologically confirmed to be advanced NSCLC. The inclusion criteria for our study were as follows: 1) patients were pathologically or radiologically confirmed to be stage IIIB or IV inoperable NSCLC; 2) patients were aged at the range of 18 and 75 years old; 3) the Eastern Cooperative Oncology Group (ECOG) of patients were at the range of 0~2. Patients were excluded when they had severe complications, such as cardiovascular and pulmonary diseases, bone marrow suppression, liver and renal dysfunctions, organ failure and brain metastasis as well as previous chemotherapy history. Patients who were pregnant and had a second primary tumor were also excluded.

All patients signed written informed consent forms to participate in clinical trial. The study protocol was approved by ethics committees of the Xinxiang Central Hospital.

***Treatment*** **: **All patients received standard first-line gemcitabine-cisplatin regimens for 4–6 cycles (21 days per cycle). If patients showed disease progression or severe adverse effects, the chemotherapy regimens were adjusted. All the patients received at least 4–6 cycles of treatment, and the follow-up was accomplished until either death or the end of the study.

Responses to chemotherapy were assessed according to WHO Response Evaluation Criteria in Solid Tumors guide, and classified into complete response, partial response, stable disease and progressive disease. Good response was defined by complete response and partial response. Poor response was defined by stable disease and progressive disease. Chest X-rays and computed tomography (CT) scans were taken to assess whether patients showed progressive disease. Survival analysis was conducted by overall survival (OS), which was calculated from the date of assignment to either the date of death or the date of last follow-up.

***RNA isolation and cDNA synthesis*** **: **5 mL peripheral venous blood samples were collected from each patient when they enrolled into this study. All the blood samples were kept in 2 mL EDTA anticoagulant tubes and stored at -70ºC until use. The total RNA was isolated from blood with an EZNA Blood RNA Mini Kit (Omega, Berkeley, CA, USA) according to manufacture’s instructions. The total extracted RNA sample was kept at -80ºC, and cDNA of ERCC1, RRM1 and TUBB3 was isolated by a fluorescence-based real-time detection method. The relative amounts of ERCC1, RRM1 and TUBB3 cDNA were evaluated in a 20 μL reaction volume, which included 50 ng genomic DNA, 200 μM dNTP and 2.5 U Taq DNA polymerase (Promega Corporation, Madison, WI, USA) as well as 200 μM primers. The expression of ERCC1, RRM1 and TUBB3 was performed using PCR, which was conducted with an initial denaturation at 94C for three minutes, followed by 35 cycles of denaturation at 94C for 30 s, reduction to the annealing temperature (64C) for 30 s, and then elongation at 72C for one minute. For quality control, a random sample of 10% cases and control subjects were selected to perform genotyping, and the reproducibility was 100%.

***Statistical analysis:*** The data were analyzed using SPSS software version 16.0 (SPSS Inc., Chicago, IL, USA). Continuous variables were showed by mean ± SD, and categorical variables were showed by frequency and percentage. The correlation of gene expression ERCC1, RRM1 and TUBB3 with different tumor response were analyzed chi-square test, and expressed by ORs and their 95% CIs. The Cox regression was used to analyze the correlation between ERCC1, RRM1 and TUBB3 expression and OS of NSCLC, and expressed by hazards ratios and 95% CIs. Association between gene expression and clinical outcome of NSCLC was analyzed by chi-square test, and expressed by ORs and their 95% CIs. All tests were 2-sided, and a significance level of P < 0.05 was used. 

## RESULTS

***Patients’ characteristics*** **: **From January 2008 to December 2009, 337 patients were collected in our study, and 305 patients agreed to participate.The patients’ characteristics of enrolled NSCLC patients are shown in Table-I.

Of the 305 patients, the median age of enrolled patients was 63.4 years, and ranged from 32.5–76.3 years. 209 patients were males and 99 were females. The histology of 160 patients were adenocarcinoma (52.46%), and 105 were squamous cell carcinoma (34.43%). 178 (58.36%) patients were at the TNM stage IV. 191(62.62%) patients at the Eastern Cooperative Oncology Group performance status of 1~2.

All the patients were followed up until December 2012, and the follow-up period was at the range of 48 and 60 months. 175 patients showed good response, and 130 patients showed poor response to chemotherapy. 126 patients died during the follow-up period (Between January 2008 and December 2012). The median OS time was 18.7 months (range, 1–60 months).

Expression levels of ERCC1, RRM1 and TUBB3 mRNA were calculated by comparing with levels of β-actin. The median levels of ERCC1, RRM1 and TUBB3 mRNA were 0.53±0.13, 0.31±0.15 and 0.18±0.16, respectively. The ERCC1, RRM1 and TUBB3 mRNA expression level was divided into high and low groups according to the median level. By chi-square test, we found that patients with low ERCC1 expression showed a significantly higher rate of good tumor response, and the adjusted OR (95% CI) was 2.16(1.32-3.45) (Table-II). However, we did not find significant association between RRM1 and TUBB3 mRNA expression and tumor response in NSCLC patients.

The associations between ERCC1, RRM1 and TUBB3 mRNA expression level and overall survival were analyzed using a Cox regression analysis. Our study showed that low ERCC1 expression level were correlated with longer overall survival of NSCLC patients when compared with high expression of ERCC1 mRNA, and the adjusted HR (95% CI) was 2.15 (1.26–3.35) (Table-III). However, we did not find significant association between RRM1 and TUBB3 mRNA expression and overall survival of NSCLC patients.

We also analyzed the association between ERCC1, RRM1 and TUBB3 mRNA expression level and histology, stage and ECOG performance stage of NSCLC, but we did not find significant association between them.

## DISCUSSION

It is generally known that traditional selection of chemotherapy regimen could improve response and survival of cancer patients, and personalized chemotherapy in NSCLC patients based on molecular characteristics can improve the overall response rate to chemotherapy and clinical outcome of cancer patients. Our study assessed the association between ERCC1, RRM1 and TUBB3 mRNA expression and response to chemotherapy and overall survival in advanced NSCLC patients. Our study suggests that ERCC1 expression can influence the tumor response and overall survival in NSCLC patients. The ERCC1 mRNA expression can be useful as prognostic markers in NSCLC patients, and can be helpful in designing personalized therapy.

ERCC1 is an important DNA damage repair gene, and it encodes the 5’-endonuclease of the NER complex. Previous study reported that high ERCC1 expression is associated with cisplatin resistance phenotype.^14^ Cisplatin-based chemotherapy can induce cytotoxicity of cancer cells through forming adducts that cause DNA cross-links. However, the NER complex can identify and remove these adducts, and thus cause resistance to platinum agents. Several studies have reported that ERCC1 expression can be an independent prognostic factor of cancer patients receiving chemotherapy.^14^^-^^17^ Li et al. reported that drug resistance to cisplatin in gastric carcinoma is correlated with increased expression of ERCC1.^15^ Muallem et al. reported that the low levels of ERCC1 expression was not associated with unfavorable outcomes of patients with locally advanced cervical cancer and ovarian cancer.^14^^,^^16^

**Table-I:** Demographic and clinical characteristics of included NSCLC in our study.

**Characteristic**	**Number** **N = 305**	**%**
Mean age (range), years	63.4 (32.5–76.3)	
Sex		
Female	99	32.46
Male	206	67.54
Smoker		
Never	141	46.23
Ever	164	53.77
Histology		
Adenocarcinoma	160	52.46
Squamous cell carcinoma	105	34.43
Mixed NSCLC	40	13.11
Stage		
IIIB	127	41.64
IV	178	58.36
ECOG performance stage		
0	114	37.38
1-2	191	62.62

***Abbreviations:*** ECOG, Eastern Cooperative Oncology Group; NSCLC, non-small cell lung cancer.

**Table-II:** Association between ERCC1, RRM1 and TUBB3 mRNA expression level and response to chemotherapy.

	**Good response**	**Poor response**	**OR (95% CI)**	**P-value**	**Adjusted OR (95% CI)**	**P-value**
	**N = 175**	**N = 130**				
ERCC1						
High	75	77	1.0 (Ref.)	-	1.0 (Ref.)	-
Low	100	53	1.94(1.19-3.15)	0.005	2.16(1.32-3.45)	<0.001
RRM1						
High	84	68	1.0 (Ref.)	-	1.0 (Ref.)	-
Low	91	62	1.19(0.74-1.92)	0.46	1.23(0.81-2.05)	0.32
TUBB3						
High	82	70	1.0 (Ref.)	-	1.0 (Ref.)	-
Low	93	60	1.32(0.82-2.14)	0.23	1.46(0.86-2.32)	0.28

***Abbreviations: ***ERCC1, excision repair cross-complementing group 1;

RRM1, ribonucleotide reductase subunit M1

**Table-III:** ERCC1, RRM1 and TUBB3 mRNA expression and overall survival of NSCLC patients

	**Deaths**	**Alive**	**Median OS (months)**	**HR (95% CI)**	**P-value**	**Adjusted HR (95% CI)**	**P-value**
	**N = 126**	**N=179**					
ERCC1							
High	51	101	21.2	1.0(Ref.)	-	1.0 (Ref.)	-
Low	75	78	32.7	1.91 (1.17–3.11)	0.006	2.15 (1.26–3.35)	<0.001
RRM1							
High	61	91	26.7	1.0 (Ref.)	-	1.0 (Ref.)	-
Low	65	88	31.5	1.11 (0.68–1.78)	0.68	1.26 (0.76–1.89)	0.42
TUBB3							
High	59	93	24.9	1.0 (Ref.)	-	1.0 (Ref.)	-
Low	67	86	32.4	1.23(0.76-1.99)	0.38	1.36(0.88-2.42)	0.24

***Abbreviations: ***CI, confidence interval; ERCC1, excision repair cross-complementing group 1;

HR, hazard ratio; NSCLC, non-small cell lung cancer; OS, overall survival;

RRM1, ribonucleotide reductase subunit M1.

For NSCLC, there were five studies which reported the association between ERCC1 mRNA expression and clinical outcome of non-small cell lung cancer treated with chemotherapy.^18^^-^^23^ Qin et al. reported that low ERCC1 mRNA expressions are correlated better PFS and OS in advanced NSCLC patients treated with chemotherapy.^18^ Zhang et al. reported that ERCC1 protein levels were significantly associated with non-small cell lung cancer.^19^ Yu et al. reported that ERCC1 mRNA levels were higher in metastatic adenocarcinoma NSCLC when compared with patients with non-metastatic disease.^21^ Our study also reported the similar results with previous ones.^18^^,^^20^^-^^23^ However, two previous studies reported inconsistent results.^17^^-^^24^ Ozdemir et al. reported that ERCC1 expression has no effect on the survival or treatment response to chemotherapy in advanced NSCLC.^17^ Tantraworasin et al. conducted a cohort study in 247 patients with completely resected advanced NSCLC, and found that ERCC1 expression can not predict the tumor recurrence and overall survival in advanced NSCLC patients.^24^ The discrepancies of these results may be caused by different populations, study design, sample sizes, and genetic distributions. Future studies are warranted to confirm the role of ERCC1 expression on the prognostic of NSCLC.

In our study, we did not fiund the association between expression levels of RRM1 and TUBB3 and tumor response to chemotherapy and clinical outcome of advanced NSCLC. Previous studies have reported the association between RRM1 and TUBB3 expression levels and clinical outcome of NSCLC, but the results are inconsistent.^18^^,1^^21^^,^^25^^-^^29^ Therefore, further large sample and well-designed study are needed to confirm our results.

This study has two limitations. First, cases were selected from one hospital, which may not be representative of the general Chinese NSCLC patients. There was still a certain risk of selection bias since they were not a random sample of the advanced NSCLC patients, and thus our study may not well represent the real situation of advanced NSCLC patients. Third, other SNPs of the DNA repaired pathway genes may affect the clinical outcome of advanced NSCLC besides the ERCC1, RRM1 and TUBB3 mRNA expression. 

In conclusion, this study showed that ERCC1 mRNA expression can not affect the response to chemotherapy and clinical outcome of advanced NSCLC patients. Further studies in Chinese NSCLC patients with larger sample size need to be conducted.

## Authors Contributions:

**HWQ & XPH** designed and performed the study, and conduct statistical analysis & editing of manuscript.

**HG, CYY & YL** did data collection and manuscript writing.
